# Circulating miR-155, miR-145 and let-7c as diagnostic biomarkers of the coronary artery disease

**DOI:** 10.1038/srep42916

**Published:** 2017-02-16

**Authors:** Julien Faccini, Jean-Bernard Ruidavets, Pierre Cordelier, Frédéric Martins, Jean-José Maoret, Vanina Bongard, Jean Ferrières, Jérôme Roncalli, Meyer Elbaz, Cécile Vindis

**Affiliations:** 1 INSERM UMR-1048, Institute of Metabolic and Cardiovascular Diseases, Toulouse, France; 2 Toulouse Paul Sabatier University, Toulouse, France; 3 CHU Toulouse, Department of Cardiology, Toulouse, France; 4 INSERM UMR-1027, Epidémiologie et Analyses en Santé publique, Toulouse, France; 5 INSERM UMR-1037, Cancer Research Center of Toulouse, France; 6 PlateformeGeT, Toulouse, France

## Abstract

Coronary artery disease (CAD) is the most prevalent cause of mortality and morbidity worldwide and the number of individuals at risk is increasing. To better manage cardiovascular diseases, improved tools for risk prediction including the identification of novel accurate biomarkers are needed. MicroRNA (miRNA) are essential post-transcriptional modulators of gene expression leading to mRNA suppression or translational repression. Specific expression profiles of circulating miRNA have emerged as potential noninvasive diagnostic biomarkers of diseases. The aim of this study was to identify the potential diagnostic value of circulating miRNA with CAD. Circulating miR-145, miR-155, miR-92a and let-7c were selected and validated by quantitative PCR in 69 patients with CAD and 30 control subjects from the cross-sectional study GENES. The expression of miR-145, miR-155 and let-7c showed significantly reduced expression in patients with CAD compared to controls. Multivariate logistic regression analysis revealed that low levels of circulating let-7c, miR-145 and miR-155 were associated with CAD. Receiver operating curves analysis showed that let-7c, miR-145 or miR-155 were powerful markers for detecting CAD. Furthermore, we demonstrated that the combination of the three circulating miRNA managed to deliver a specific signature for diagnosing CAD.

Coronary artery disease (CAD) is still the most prevalent cause of mortality and morbidity worldwide. Despite recent advances in diagnosis, treatment and prognosis of cardiovascular diseases, there is still a clinical need to identify novel diagnostic and prognostic biomarkers that pave the way for new therapeutic interventions. Indeed, it is challenging to improve the conventional cardiovascular risk scores by assessing new biomarkers that will complement clinical decision-making and help to stratify patients for early preventive treatment. MicroRNA (miRNA) are a class of small (~22 nucleotides) noncoding RNA that are essential post-transcriptional modulators of gene expression that bind to the 3′ untranslated region of specific target genes, thereby leading to suppression or translational repression^1^. Accumulating evidence indicate that miRNA are critically involved in physiological or pathological processes including those relevant for the cardiovascular system^2^,^3^. The majority of miRNA are intracellular however miRNA can be secreted as micro vesicles or exosomes and apoptotic bodies into the blood circulation. MiRNA remain stable in the blood or serum, and membrane-derived vesicles or lipoproteins can carry and transport circulating miRNA. Indeed, miRNA isolated from plasma are highly stable in boiling water, prolonged room temperature incubation or repeated freeze-thawing^4^. Several studies indicate that circulating miRNA are protected from plasma ribonucleases by their carriers e.g. lipid vesicles or protein conjugates (such as Argonaute 2 or other ribonucleoproteins)^5^.

Specific expression profiles of circulating miRNA have been associated with several diseases such as cancer and cardiovascular injury therefore miRNA have emerged as potential suitable biomarkers for accurate diagnosis^6^. The aim of the present study was to investigate circulating miRNA differentially regulated between patients with CAD and control subjects and, determine their potential diagnostic value for CAD. We identify associations of miRNA as a new blood-based miRNA signature for the detection of CAD.

## Results

### Study characteristics

The baseline characteristics of the 69 patients with CAD (from the original 70 patients, one was excluded because of poor RNA quality) and 32 control subjects (from the original 35 subjects, 3 were excluded because of poor RNA quality) are summarized in Table 1. Among the metabolic markers, total cholesterol or LDL-cholesterol were lower in individuals with CAD, reflecting effects of lipid-lowering drugs in patients. However, patients with CAD displayed higher levels of triglycerides and lower HDL-cholesterol or ApoA1 concentrations. The percentage of current smokers was significantly higher in patients with CAD compared to control subjects.

### Screening and validation of candidate miRNA by RT-qPCR

**Figure 1:**
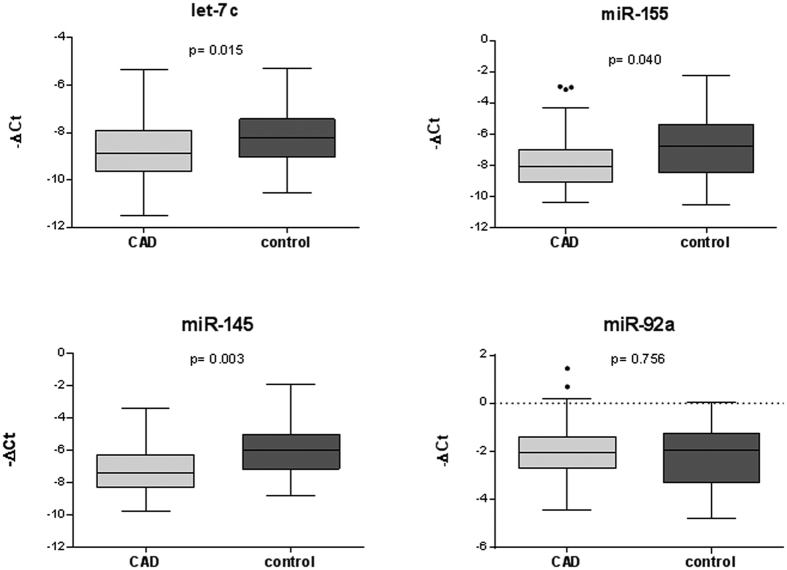
Plasma miRNA levels in the validation population. The box plots show the expression levels of let-7c, miR-155, miR-145 and miR-92a measured by quantitative real-time polymerase chain reaction (qRT-PCR) in patients with CAD (n = 69) and control subjects (n = 32). The relative miRNA expression levels were normalized to cel-miR-39 and calculated by −ΔCt.

**Figure 2:**
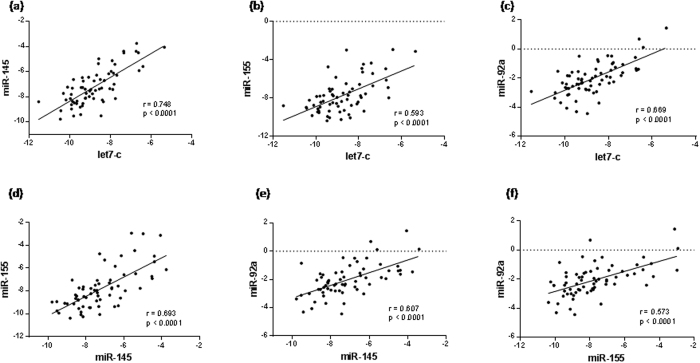
Spearman correlations between the circulating miRNA in patients with CAD. The scatter plots show the marked correlation in the −ΔCt value between (**a**) let-7c and miR-145 and (**b**) miR-155 and (**c**) miR-92a, and (**d**) between miR-155 and miR-145, and (**e**) between miR-92a and miR-145 and (**f**) miR-155 in the population (n = 69) with CAD.

**Figure 3:**
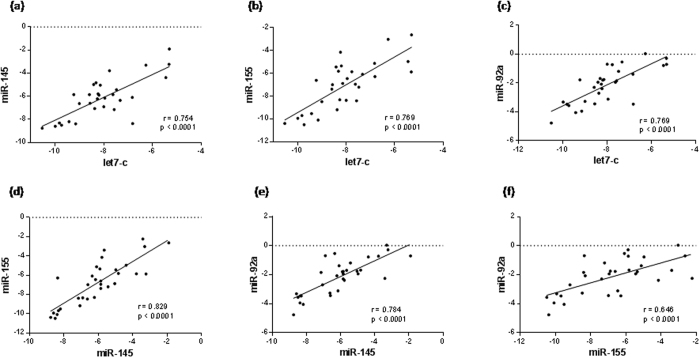
Spearman correlations between circulating miRNA in control subjects. The scatter plots show the marked correlation in the −ΔCt value between (**a**) let-7c and miR-145 and (**b**) miR-155 and (**c**) miR-92a, and (**d**) between miR-155 and miR-145, and (**e**) between miR-92a and miR-145 and (**f**) miR-155 in the control (n = 32) population.

The first phase of the work was the validation of the good quality of the human plasma samples for detecting circulating miRNA. A miRNA microarray profile using chip-based digital PCR was first carried out using EDTA plasma RNA isolated from age matched patients with CAD (n = 3) and control subjects (n = 3) of both studied group. Data obtained from microarray analysis revealed a number of miRNA that were differentially regulated in the plasma of patients with CAD compared to control subjects. Supplemental data Figure S1 shows the −ΔCt values and fold changes of each detected miRNA in both control and CAD groups miRNA that were up- or down-regulated. Based on array results and literature mining we selected four miRNA candidates: an inflammatory cell-related miR-155, a vascular smooth muscle cell (SMC)-related miR-145, an endothelial cell (EC)-related miR-92 and a cell differentiation-related miRNA let-7c. We further validated by quantitative RT-PCR the expression of the four miRNA in the whole population (including 69 patients with CAD and 32 control subjects). As shown in Fig. 1, we measured a significant decreased in expression levels of let-7c (p = 0.015), miR-155 (p = 0.040) and miR-145 (p = 0.003) in patients with CAD compared with control subjects. Although circulating miR-92a levels are correlated with CAD^7^,^8^,^9^ no significant difference was found in the expression level of miR-92a (p = 0.756) between patients with CAD and control subjects. Clustered and homologous miRNA could show similar or consistent patterns of deregulation, so to ascertain whether these miRNAs are related or not to each other we performed correlation analysis of their expression. Interestingly, we found a striking correlation measured by Spearman coefficient between let-7c, miR-155, miR-145 and miR-92a expression levels in the group of patients with CAD (Fig. 2a–f) and in the control group (Fig. 3a–f). However, the genomic context could not be involved in these marked correlations since as shown on the miRBase and HGNC (Human Genome Organisation, Gene Nomenclature Committee) websites, miR-145 is localized on chromosome 5 (5q32), miR-92a is on chromosome 13 (13q31.3), let-7c and miR-155 are localized on the same chromosome 21 but in different region 21q21.3 and 21q21.1, respectively.

### Correlation, multivariate analysis and diagnostic accuracy of the candidate miRNA

Spearman rank correlation coefficients between the levels of plasma let-7c, miR-145, miR-155 and miR-92a and metabolic parameters or cardiovascular risk markers were calculated (Table 2). In patients with CAD we found a significant positive correlation of let-7c (r = 0.318, p = 0.009), miR-145 (r = 0.433, p < 0.001), miR-155 (r = 0.303, p = 0.013) and miR-92a (r = 0.364, p = 0.002) levels with total cholesterol, whereas no correlation was found in control subjects. A significant positive correlation was found between let-7c (r = 0.284, p = 0.025), miR-145 (r = 0.418, p < 0.001) and miR-92a (r = 0.330, p = 0.008) levels with atherogenic LDL-cholesterol, whereas no correlation was found in control subjects. The pro-atherogenic apoB levels were correlated with miR-145 (r = 0.379, p < 0.001), miR-155 (r = 0.262, p = 0.032) and miR-92a (r = 0.264, p = 0.029) levels in patients with CAD whereas no correlation was found in control subjects. Triglycerides were significantly correlated with miR-155 levels in patients with CAD (r = 0.386, p = 0.001) and control subjects (r = 0.360, p = 0.043), with let-7c (r = 0.270, p = 0.027) in patients with CAD. No statistical association could be found between the four miRNA and HDL-cholesterol or ApoA1 in patients with CAD and control subjects. Age was negatively correlated with let-7c (r = −0.240, p = 0.051) and miR-145 (r = −0.374, p = 0.002) levels in patients with CAD whereas miR-155 was associated with age in control patients. Blood glucose was negatively correlated with miR-92a (r = −0.289, p = 0.016) levels in patients with CAD whereas let-7c (r = 0.353, p = 0.047) and miR-155 (r = 0.491, p = 0.004) levels were associated with blood glucose in control subjects.

**Figure 4:**
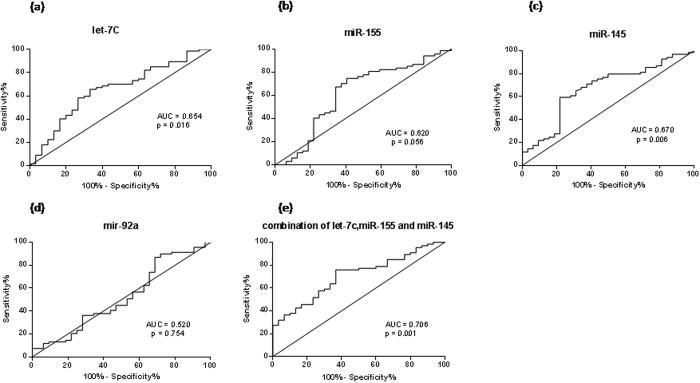
Receiver operating characteristic (ROC) curves analysis of let-7c, miR-155, miR-145 and miR-92a for detecting CAD. The areas under the curves (AUC) are 0.654 (95% CI: 0.536–0.773, p = 0.016) for let-7c (**a**), 0.620 (95% CI: 0.491–0.747, p = 0.056) for miR-155 (**b**), 0.670 (95% CI: 0.556–0.785, p = 0.006) for miR-145 (**c**), 0.520 (95% CI: 0.393–0.646 p = 0.754) for miR-92a (**d**) and 0.706 (95% CI: 0.600–0.811 p = 0.001) for the combination of let-7c, miR-155 and miR-145 (**e**). CI, confidence interval.

Univariate logistic regression analysis revealed that let-7c (p = 0.006) and miR-145 (p = 0.014) were significantly associated with the risk of CAD, miR-155 showed a tendency for association with the risk of CAD (p = 0.063) and miR-92a showed no association (Table 3). These variables were entered into a backward, stepwise, multivariate logistic regression model that includes lipid parameters, smoking and medication. Even after adjustment the low levels of circulating let-7c, miR-145 and miR-155 were still independently associated with CAD (Table 3). To investigate the possibility that these circulating miRNA may serve as new and potential biomarkers for CAD, a ROC curve analysis was performed to evaluate the diagnostic accuracy of each selected miRNA. As shown in Fig. 4, the area under the curve (AUC) was 0.654 (p = 0.016) for let-7c, 0.620 (p = 0.056) for miR-155 and 0.670 (p = 0.006) for miR-145 to predict CAD whereas miR-92a is still not specific for diagnosing CAD with an AUC of 0.520 (p = 0.754). The cut-off values of each single miRNA between patients with CAD and healthy subjects were >0.691 (65.67% sensitivity, 66.67% specificity) for let-7c, >0.720 (59.42% sensitivity, 78.12% specificity) for miR-145 and >0.660 (74.63% sensitivity, 59.38% specificity) for miR-155. Interestingly the combination of let-7c, miR-155 and miR-145 resulted in a much higher AUC value of 0.708 (95% CI: 0.600–0.811, p = 0.001), increasing the diagnostic power to a 75.76% sensitivity and a 63.33% specificity thus leading to a sensitive and independent signature for the prediction of CAD.

## Discussion

Based on their tissue-specific expression, rapid release into the circulation and remarkable stability in plasma, circulating miRNA are currently explored for their potential as biomarkers for diagnosis or prognosis of cardiovascular diseases. Indeed, sampling of circulating plasma miRNA may represent a potential new approach for rapid and non invasive diagnostic screening using RT-qPCR^6^. Our study reports for the first time a novel signature of three circulating miRNA let-7c, miR-155 and miR-145 allowing to distinguish patients with CAD from non-CAD control subjects. In accordance with the current literature^4^, our results showed that circulating miRNA are differentially regulated in the plasma of patients with CAD compared to control subjects. Plasma levels of vascular SMC-enriched miR-145 and the inflammatory cell-related miR-155 were found to be significantly down-regulated in patients with CAD compared to the control subjects. Although plasma levels of EC-enriched miR-92a were up-regulated in patients with CAD in the miRNA profiling experiment, this increase did not attain statistical significance in the validation cohort. Interestingly, in patients with CAD we identified for the first time reduced plasma levels of let-7c, a member of the let-7 family which has been involved in heart development and cardiovascular differentiation^10^,^11^. Multivariate analysis showed that low levels of circulating let-7c, miR-145 and miR-155 were associated with CAD and our receiver operating curves revealed that let-7c, miR-145 or miR-155 may be potential markers for CAD. Furthermore, the combination of the three miRNAs managed to deliver an increased diagnostic power with the AUC of 0.708 (95% confidence interval 0.600–0.811). Because the plasma levels of let-7c, miR-155 and miR-145 were found to be lower in patients with CAD when compared with control subjects, we propose that the downregulation of these miRNAs plays a critical role in the pathogenesis of CAD and perhaps these miRNAs displayed reduced expression, enhanced degradation, or are taken up in diseased vessels. As discussed in previous reports^7^, changes in miRNA between patients with CAD and healthy subjects could be also, in part, due to the mean difference in age between the two groups. It is quite unlikely in our study since the studied population was matched on age. Moreover, miRNA are responsive to therapeutic interventions^12^,^13^ such as statins or anti-platelet agents that could influence the levels of miRNA and too often adjustment for medication is lacking. Importantly, although medication can be a confounding factor, our results showed that even after adjustment for the use of medication the levels of the three circulating miRNA were still independently associated with CAD, thus supporting the significance of these miRNA in CAD.

Prior studies have shown that the miR-143/145 cluster plays a critical role in vascular SMC phenotypic modulation in normal and abnormal vascular pathologies^14^,^15^. MiR-145 is exclusively expressed in vascular SMC and is downregulated in animal models of vascular injury^16^, in human plaque atherosclerosis and aneurysms^14^. Mice deficient for miR-145 have a thinner medial layer in arteries and decreased blood pressure indicating severely disturbed SMC homeostasis^14^. Conversely, overexpression of mir-145 in SMC reduced neointima formation in rat carotid arteries after balloon injury^16^ and atherosclerotic plaque burden in ApoE−/− mice^17^. The mechanisms involved in miR-145 control of vascular SMC phenotype include target genes such as myocardin, Kruppel-like factor (KLF) 4 and KLF5, angiotensin-converting enzyme or calmodulin kinase (CAMK) IIδ^18^. In addition, the cholesterol transporter ABCA1 was identified as a target of miR-145^19^ and the absence of miR-145 in mice results in a marked decrease of circulating cholesterol (VLDL and LDL) compared with Ldlr^−/−^ mice^19^. Our results are in agreement with these data since we found that miR-145 is positively correlated to total cholesterol, LDL-cholesterol and ApoB in patients with CAD. Although lower plasma miR-145 levels have shown to be associated with the severity of CAD quantified by the Gensini score^20^, we could not identify any relation between plasma miR-145 levels and severity scores (data not shown). Altogether these results and our current studies emphasize the significance of miRNA-145 as a biomarker of CAD and support that manipulation of miRNA-145 expression may offer new therapeutic approach for attenuating atherosclerosis.

MiR-155 has recently emerged as a novel component of inflammatory signal transduction in atherosclerosis and is considered as a mechanosensitive athero-miRNA^2^. Expression of miR-155 is up-regulated in atherosclerotic lesions of human and mice, predominantly in pro-inflammatory macrophages and SMC where it targets BCL6 (B-cell lymphoma 6) and HMGB1 (High mobility group box 1)^21^. In the present study, plasma miR-155 was positively correlated with total cholesterol in patients with CAD which supports previous data showing that overloading with free cholesterol strongly upregulates miR-155 levels in macrophages^22^. Recent work demonstrated that miR-155 exerts both anti-angiogenic function via suppression of AT1R (Angiotensin II Receptor type 1) and pro-arteriogenic function via the modulation of SOCS1 (suppressor of cytokine signaling protein 1) in mouse model of ischemia^23^. MiR-155 is significantly upregulated in human abdominal aortic aneurysm^24^ and an increased transcoronary gradient of miR-155 is associated with the extent of vulnerable plaques^25^. Interestingly, in these two studies systemic levels of miR-155 are not correlated with its tissue expression and display decreased expression compared to healthy subjects as we observed in our study. Inverse association was also found between severity of coronary stenotic lesions and plasma miR-155^13^. One explanation for this discrepancy could be the uptake of circulating miRNA by recipient cells^26^ or even by atherosclerotic lesions^25^,^27^ that causes a decrease of circulating miRNA in CAD patients.

Recently, the let-7 family has been discovered to play important roles both in cardiovascular biology and diseases where let-7 members participates in cardiovascular differentiation of embryonic stem cells^10^. Currently, the inhibition of let-7c attenuates myocardial remodeling and prevents deterioration of cardiac functions post-infarction in mice as a result of increased expression of pluripotency-associated genes Oct4 and Sox2^28^. Conversely, a vascular function of let-7c has been described; for instance, its overexpression enhances apoptosis in endothelial cells through the inhibition of Bcl-xl a direct target of let-7csuggesting a pro-atherosclerotic role of let-7c^29^. Moreover, let-7c regulates cellular functions associated with macrophage phenotypes by promoting M2 polarization and suppressing M1 activation through the regulation of the transcription factor C/EBP-δ^30^. Although let-7c positively correlated with total cholesterol, LDL cholesterol and triglycerides in patients with CAD during this study, further investigations are needed to confirm its role in lipoprotein metabolism. Interestingly, our study is the first to show that decreased circulating let-7c is associated with CAD thus we can speculate that dynamic changes in the expression of let-7c could reflect pathological states.

Several limitations have to be taken into account. Although the sample size studied had enough statistical power to detect significance for all of the analyses, including a second independent cohort for validation would be preferred. However, the studied population is a high-risk population reflecting residual cardiovascular risk and the participants represent a sample of a prospective case-control study very well documented and followed-up^31^,^32^. A large scale multicenter study is warranted to further validate the performance of the association of miR-145, miR-155 and let-7c as a potential blood-based signature in CAD. Indeed, the investigation of the incremental value of circulating miRNA signature when added to a clinical risk score might be clinically relevant. Additional studies are necessary to unravel the mechanisms underlying the association between lower circulating miR-145, miR-155 and let-7c levels and CAD diagnosis but this is beyond the scope of the present report.

In conclusion, we demonstrate for the first time a unique signature of circulating miRNA for sensitive and specific diagnosis of CAD that could be translated into non invasive blood-based biomarker panels for patients. Considering that miRNA are active molecules, our study also suggests that miR-145, miR-155 and let-7c may be instrumental in CAD pathology and could represent new targets for therapeutic interventions.

## Methods

### Study Population

The studied population belongs to the cross-sectional study “Génétique et Environnement en Europe du Sud” (GENES) that was designed to assess the role of genetic, biological and environmental determinants in the occurrence of coronary heart disease^31^,^32^,^33^. The study protocol has been conducted according to the principles of the Declaration of Helsinki. The protocol was endorsed by the Scientific Council of the Toulouse University Hospital and was approved by the “Comité Consultatif pour la Protection des Personnes se prêtant à la Recherche Biomédicale” (Advisory Committee regarding protection of persons involved in medical investigation, Comité Toulouse/Sud-Ouest #1) file number 1-99-48. The biological sample collection was declared to the French Ministry of Research and to the Regional Health Agency under number DC-2008-403 #1. Information was provided about the objectives of the study, and participants provided written informed consent. As previously described^33^ case cohort included stable male CAD patients living in the Toulouse area (South-western France), aged between 45 and 74. From 2001 to 2004, we have prospectively recruited 834 male patients aged between 45 and 74, living in the Toulouse area (South-western, France), admitted in the department of Cardiology of the Toulouse University Hospital, and referred for evaluation and management of stable CAD.

CAD was defined by a previous history of acute coronary syndrome, a previous history of coronary artery revascularization, a documented myocardial ischemia, a stable angina, or the presence at the coronary angiography of a coronary stenosis of ≥50% of luminal narrowing. These CAD patients are clinically stable and did not present recent acute coronary event prior to one month before the hospital admittance for the study. During the same period, the male control cohort of the GENES study was randomly constituted using electoral rolls representative of the general population. We obtained by phone call the participation agreement of 820 males. They were aged between 45–74 years and were living in the same area (Toulouse, South-western, France). Personal history of coronary heart disease was systematically checked using a specific questionnaire recording information on previous hospitalizations and medical treatments currently taken (a copy of the last medical prescription was requested). An electrocardiogram was performed during medical examination and was carefully reviewed by a cardiologist for abnormalities. All these information were used to ensure that the subjects were not suffering from coronary heart disease. A subject classified having coronary heart disease was not considered and selected as control. Stratification by 10-age group (4 decadals age groups ranging from 35 to 74 years: 35y–44y; 45y–54y; 55y–64y and 65y–74y) was employed to approximately match the age distribution between controls and cases. Controls and cases underwent medical examination in the same health center and during the same period, including clinical and anthropometric measurements and completion of a questionnaire. For our current study, 70 patients from the GENES case cohort were randomly selected. Then, 35 control subjects (from the GENES control cohort) were matched to cases with respect to age, HTA, diabetes and overall mortality with a ratio of 2 cases for one control. The 3 control subjects and the 3 CAD patients used in the feasibility phase belong to the studied population and were age matched. Taking into account an odds ratio of 0.50, 0.55 and 0.60 at the 0.05 significance level with a two-sided test and a sample size of 105 subjects corresponded to the whole studied population, the power varies between 90%, 80% and 70% respectively. The sample size study of 105 subjects seems to be a sufficient number to put into evidence a significant difference (alpha = 0.05) between cases and controls for a majority of miRNA studied.

### Data Collection

Age, socio economic variables and information on cardiovascular risk factors were collected through standardized face-to-face interviews, performed by a single physician. Dyslipidemia was defined as treatment with drugs or fasting serum total cholesterol ≥2.40 g/L. Hypertension was defined as treatment with drugs or systolic blood pressure ≥160 mmHg or diastolic blood pressure ≥95 mmHg. Diabetes was defined as treatment with drugs or fasting blood glucose ≥7.8 mmol/L. Smoking status was classified as current smokers, former smokers having quit tobacco for more than 3 months and non-smokers. In patients, medications at discharge were also considered. Anthropometrical measurements included waist circumference, height, body weight and body mass index (BMI) was calculated (kg/m^2^). Blood pressure and resting heart rate were measured after ≥5 min rest with an automatic sphygmomanometer. Two measurements were performed and average values were recorded.

### Biological Measurements

Blood was collected after an overnight fast. Blood glucose, triglycerides, total cholesterol and HDL-cholesterol were assayed with enzymatic reagents on an automated analyzer (Hitachi 912, Roche diagnostics, Rotkreuz, Switzerland). LDL-cholesterol was calculated using the Friedwald formula. CRP and γ GlutamylTransferase were also analyzed with an automated analyzer (Hitachi 912, Roche-Diagnostics, Rotkreuz, Switzerland). ApoA-I, apo-B and lipoprotein (a) (LpA) were determined by first order precipitation in an automated analyzer (Hitachi 912, Roche-Diagnostics, Rotkreuz, Switzerland). LpA-I was also determined by electro-immunoassay (Sebia, Evry, France).

### Total RNA isolation and quality control

Total RNA was extracted from 100 μL of EDTA plasma using the miRNeasy serum/plasma kit (Qiagen) according to the manufacturer’s instructions. A 5 μL aliquot of 1 nM synthetic *Caenorhabditis elegans* miR-39-3p (cel-miR-39-3p) miRVana miRNA Mimic (PN 4464066) was spiked into each plasma sample after addition of QIAzol to monitor the efficiency of miRNA recovery and to normalize miRNA expression in the subsequent real time PCR as described previously^7^. The quality and quantity of RNA were measured with a NanoDrop spectrophotometer (NanoDrop Technologies, Wilmington, DE, USA).

### MicroRNA expression profiling

The different miRNA profiles of 754 miRNA between CAD patients and control subjects were determined using the Life Technologies TaqMan Open Array Human MicroRNA panel^34^. Reverse transcription (RT) and preamplification were performed on all samples using Megaplex Primer Pools A and B v3.0 (PN 4444750) with the recommended protocol (LTC publication PN 4470935 Rev. C) for TaqMan Open Array microRNA panel (PN 4461104). The cDNA was prepared with 3 μL of total RNA per sample using TaqMan MicroRNA Reverse Transcription Kit (PN 4366596) and Megaplex RT Primer Pools in 7.5 μL final volume. The RT reaction was thermal cycled (2 min at 16 °C, 1 min at 42 °C, 1 s at 50 °C, for 40 cycles) and the enzyme inactivated at 85 °C for 5 min. Preamplification was performed using 2.5 μL of the RT reaction and combined with the matching Megaplex PreAmp Primer Pool and TaqMan PreAmp Master Mix (PN 4391128) in a final volume of 25 μL. Preamplification was run using the following cycling conditions: 10 min at 95 °C; 2 min at 55 °C; 2 min at 72 °C; 15 s at 95 °C, 4 min at 60 °C for 12 cycles; 99 °C for 10 min. The preamplification product was diluted 1:40 in TE and then diluted 1:2 with TaqMan Open Array Real-Time PCR Master Mix (PN 4462164). Aliquots of 5 μL of the diluted reaction mixture for each sample were placed into eight wells on an Open Array 384 well sample plate where each well corresponds to one subarray on Open Array Plate. Open Array plates were automatically loaded by AccuFill using a standard AccuFill method (Open Array AccuFill System User Guide PN 4456986). Data analysis was carried out using QuantStudio 12 K Flex, v1.2.2 and Expression Suite, v1.0.3, cut off values of Ct considered as eligible was 35 and global mean normalization was used to calculate relative fold changes.

### miRNA validation

The expression of the selected plasma miRNA was quantified with RT-qPCR. RT and pre-amplification was performed on all samples using custom RT primer pool and custom preamp primer pool (PN 4427975) with the recommended protocol (LTC publication PN 4465407 Rev. B). The cDNA was prepared with 3 μL of total RNA per sample using TaqMan MicroRNA Reverse Transcription Kit and custom primer pool in 15 μL final volume. The RT reaction was thermal cycled (30 min at 16 °C, 30 min at 42 °C) and the enzyme inactivated at 85 °C for 5 min. Pre-amplification was performed using 2.5 μL of the RT reaction was combined with TaqMan PreAmp Master Mix and the custom preamp primer pool in a final volume of 25 μland was run under the same cycling conditions as the TaqMan Open Array, above, with one modification; 15 cycles of pre-amplification were performed instead of 12. The pre-amplification product was diluted 1:8 in TE then diluted 1:100 in 1× TaqMan Universal Master Mix II (PN 4440040) before loading on 96-wells plate. The 96-wells plates were sealed, spun and then run on a StepOne Plus Real Time PCR system using universal cycling conditions (10 min at 95 °C; 15 s at 95 °C, 1 min at 60 °C, 40 cycles). Normalization to cel-miR-39 was applied as described^7^,^35^, and values were expressed as −ΔCt. Data were analyzed using the Applied Biosystems analysis Software v3.1 on the Thermo Fisher cloud.

### Statistical analysis

Data are presented as means and standard deviations for quantitative variables and percentages for categorical variables. The Chi^2^ test was used to compare the distribution of qualitative variables between cases and controls. When basic assumptions were not satisfied, data were subjected to Fisher’s exact test. Mean values of quantitative variables were compared by Student’s *t*-test. Shapiro-Wilk’s and Levene’s tests were used to test the normality of distribution of residuals and the homogeneity of variances, respectively. When basic assumptions of Student’s *t*-test were not satisfied, a logarithmic transformation of the variables was done or a Wilcoxon-Mann-Whitney test was performed. Spearman rank correlations, with a graphical scatterplot representation, were performed to test the associations of miRNA expression levels among each other and with blood lipid parameters. Box plots were displayed for each miRNA according to case-control status and a Student’s *t*-test was made to assess mean differences. The relationships between miRNA and case-control status were assessed using logistic regression. The relationships were first tested in univariate analysis and then in multivariate analysis by adjusting for variables of the basic model including LDL-cholesterol, HDL-cholesterol, smoking, statins, ACE inhibitors, beta blockers and calcium channel blockers. To establish for each miRNA the ability to differentiate cases and controls, the receiver operating characteristics (ROC) curve analysis was used and the calculation of the standard error of the area under the curve (AUC) was done applying the Delong’s method. All tests were two-tailed at the level of significance of 0.05. All analysis was carried out using SAS software, version 9.4 (SAS Institute, Cary, NC, USA).

## Additional Information

**How to cite this article:** Faccini, J. *et al*. Circulating miR-155, miR-145 and let-7c as diagnostic biomarkers of the coronary artery disease. *Sci. Rep.* **7**, 42916; doi: 10.1038/srep42916 (2017).

**Publisher's note:** Springer Nature remains neutral with regard to jurisdictional claims in published maps and institutional affiliations.

## Supplementary Material

Supplementary Data S1

## Figures and Tables

**Table 1:** Baseline characteristics of patients with CAD and control subjects.

Variable	CAD	Control	*p-value*
	n = 69	n = 32	
Age (years)	58.4 ± 9.0	57.3 ± 11.6	0.61
BMI (kg/m^2^)	27.3 ± 4.4	26.6 ± 3.1	0.39
Waist (cm)	100.5 ± 12.4	96.8 ± 10.7	0.15
SBP (mm Hg)	136.5 ± 21.7	140.9 ± 20.7	0.34
DBP (mm Hg)	83 ± 10.6	83.2 ± 10.5	0.96
Blood glucose (mmol/L)	5.96 ± 2.04	5.98 ± 1.43	0.39*
Current smoker (%)	29	12.5	**0.02**
Past (%)	43.5	31.3	
Never (%)	27.5	56.3	
Hypertension (%)	36.2	37.5	0.91
Diabetes (%)	36.2	28.1	0.43
Triglycerides (g/L)	2.03 ± 1.36	1.17 ± 0.61	0.001**
Total cholesterol (mg/dL)	197.1 ± 44.1	217.5 ± 28.2	0.009*
LDL cholesterol (mg/dL)	117.6 ± 38.2	139.7 ± 32.3	**0.007**
HDL cholesterol (mg/dL)	41.7 ± 13.5	54.3 ± 14.0	**0.001**
ApoA1 (g/L)	1.21 ± 0.24	1.52 ± 0.26	**<0.001**
ApoB (g/L)	1.01 ± 0.27	1.02 ± 0.22	0.843
Lpa (g/L)	0.45 ± 0.44	0.37 ± 0.55	0.44
Beta blocker agents (%)	49.3	0	**0.001**
Calcium channel blockers (%)	14.5	0	0.03***
ACE inhibitors (%)	20.3	3.1	0.03***
Statins (%)	53.6	28.1	**0.02**
Fibrates (%)	8.7	9.4	0.98***
Antiplatelet agents (%)	94.2	5.8	**0.001**

Data are represented as mean ± standard deviation, bold values indicate statistical significance. ApoA1, apolipoprotein A1; ApoB, apolipoprotein B, BMI, body mass index; DBP, diastolic blood pressure; HDL, high-density lipoprotein; LDL, low-density lipoprotein; Lpa, lipoprotein (a); SBP, systolic blood pressure.

^*^Wilcoxon-Mann-Whitney test.

^**^Log transformed data.

^***^Fisher’s exact test.

**Table 2:** Relationships between let-7c, miR-155, miR-145, miR-92a and classical cardiovascular risk factors in patients with CAD and control subjects.

		CAD (n = 69)				Controls (n = 32)			
Variable		let-7c	miR-145	miR-155	miR-92a	let-7c	miR-145	miR-155	miR-92a
Age (years)	r=	**−0.240**	**−0.374**	−0.198	−0.155	0.271	0.243	**0.436**	−0.262
	*p*=	**0.051**	**0.002**	0.108	0.203	0.147	0.180	**0.013**	0.147
BMI (kg/m^2^)	r=	−0.109	−0.183	−0.080	0.009	0.083	0.056	0.241	0.044
	*p*=	0.378	0.133	0.515	0.943	0.650	0.762	0.184	0.811
Waist (cm)	r=	−0.182	−0.236	−0.137	−0.053	0.108	0.114	0.230	0.043
	*p*=	0.140	0.051	0.260	0.666	0.555	0.534	0.206	0.817
SBP (mm Hg)	r=	−0.141	−0.238	−0.171	−0.057	0.268	0.267	**0.419**	0.339
	*p*=	0.258	0.049	0.160	0.642	0.138	0.139	**0.017**	0.058
DBP (mm Hg)	r=	0.068	0.048	0.061	0.091	**0.391**	0.329	**0.379**	**0.543**
	*p*=	0.584	0.693	0.617	0.458	**0.027**	0.066	**0.033**	**0.001**
Blood glucose (mmol/L)	r=	−0.131	−0193	−0.172	**−0.289**	**0.353**	0.139	**0.491**	0.113
	*p*=	0.289	0.112	0.158	**0.016**	**0.047**	0.446	**0.004**	0.539
Triglycerides (g/L)	r=	**0.270**	0.192	**0.386**	0.139	0.234	0.234	**0.360**	0.203
	*p*=	**0.027**	0.114	**0.001**	0.255	0.214	0.197	**0.043**	0.266
Total cholesterol (mg/dL)	r=	**0.318**	**0.433**	**0.303**	**0.364**	−0.195	0.068	−0.063	−0.159
	*p*=	**0.009**	**<0.001**	**0.013**	**0.002**	0.247	0.711	0.729	0.384
LDL cholesterol (mg/dL)	r=	**0.284**	**0.418**	0.207	**0.330**	−0.343	−0.005	−0.250	−0.210
	p=	**0.025**	**<0.001**	0.106	**0.008**	0.064	0.980	0.168	0.251
HDL cholesterol (mg/dL)	r=	−0.022	0.013	−0.144	0.042	0.218	0.075	0.200	0.022
	*p*=	0.858	0.917	0.244	0.732	0.147	0.683	0.271	0.905
ApoA1 (g/L)	r=	0.015	0.019	−0.047	0.057	0.148	−0.086	0.108	−0.041
	*p*=	0.902	0.880	0.708	0.644	0.436	0.641	0.556	0.823
ApoB (g/L)	r=	0.194	**0.379**	**0.262**	**0.264**	−0.199	0.067	−0.132	−0.129
	*p*=	0.116	**0.001**	**0.032**	**0.029**	0.293	0.717	0.473	0.481
Lpa (g/L)	r=	−0.034	−0.028	0.018	−0.055	**−0.380**	−0.061	−0.197	−0.262
	*p*=	0.784	0.819	0.888	0.655	**0.038**	0.740	0.279	0.147

r = Spearman rank correlation coefficients, *p* = *p* value. Bold values indicate statistical significance. ApoA1, apolipoprotein A1; ApoB, apolipoprotein B, BMI, body mass index; DBP, diastolic blood pressure; HDL, high-density lipoprotein; LDL, low-density lipoprotein; Lpa, lipoprotein (a); SBP, systolic blood pressure.

**Table 3:** Univariate and multivariatelogistic regression analysis for the risk of CAD.

	Univariate logistic regression			Multivariate logistic regression^a^		
	OR	95% CI	*p*-value	OR	95% CI	*p-*value
let-7c	0.63	0.45–0.87	0.006	0.47	0.27–0.83	0.0092
miR-145	0.74	0.58–0.94	0.014	0.68	0.49–0.96	0.028
miR-155	0.83	0.68–1.01	0.063	0.74	0.56–0.98	0.041
miR-92a	1.14	0.79–1.64	0.496	1.06	0.66–1.71	0.81

^a^The model included LDL, HDL and smoking, statins, ACE inhibitors, beta blockers and calcium channel blockers. CI = confidence interval, OR = odds ratio. OR were given for variation of one unit of miRNA.
