# Supplementary material for: Circulating miR-155, miR-145 and let-7c as diagnostic biomarkers of the coronary artery disease
Source: Sci Rep. 2017 Feb 16;7:42916. doi: 10.1038/srep42916 (PMC5311865; doi:10.1038/srep42916)
Supplement: Supplementary Data S1 [file srep42916-s1.pdf]

Supplementary Information

Circulating miR-155, miR-145 and let-7c as diagnostic biomarkers of the coronary artery disease

Julien Faccini [1,2], Jean-Bernard Ruidavets [2,3,4], Pierre Cordelier [5], Frédéric Martins [6], Jean-José Maoret [6], Vanina Bongard [2,3], Jean Ferrières [2,3,4], Jérôme Roncalli [3], Meyer Elbaz [1,2,3] and Cécile Vindis [1,2]

[1] *INSERM UMR-1048, Institute of Metabolic and Cardiovascular Diseases, Toulouse, France;*
[2] *Toulouse Paul Sabatier University, Toulouse, France;*
[3]*CHU Toulouse, Department of Cardiology, Toulouse, France;*
[4]*INSERM UMR-1027, Epidémiologie et Analyses en Santé publique, Toulouse, France ;*
[5]*INSERM UMR-1037, Cancer Research Center of Toulouse, France;*
[5] *PlateformeGeT, Toulouse, France*

Correspondence to: Cécile Vindis, PhD

INSERM UMR-1048/I2MC

1 avenue Jean Poulhès - BP84225 - 31432 Toulouse Cedex 4 - France.

Tel. + 33 561-32-2705 - **Fax** + 33 561-32-2084

E-mail : cecile.vindis@inserm.fr

Supplemental data- **Figure S1**

Circulating miRNA detected in plasma of CAD patients and control subjects.

miRNA	CAD	SEM	Control	SEM	fold change
dme-miR-7_000268	-11,34	0,13	-6,58	2,43	0,037
hsa-let-7c_000379	-1,84	0,62	-9,63	1,85	221,552
hsa-let-7d_002283	1,44	0,51	2,65	0,96	0,433
hsa-let-7e_002406	4,67	0,05	5,32	0,26	0,637
hsa-let-7g_002282	2,61	0,22	3,86	1,08	0,420
hsa-miR-100_000437	-7,65	3,78	-9,87	1,61	4,659
hsa-miR-101_002253	-4,40	3,39	0,65	1,36	0,030
hsa-miR-103_000439	2,36	0,17	3,40	0,85	0,489
hsa-miR-106a_002169	8,29	0,19	9,25	0,90	0,517
hsa-miR-106b_000442	3,26	0,51	5,36	1,05	0,234
hsa-miR-107_000443	-11,34	0,13	-9,87	1,61	0,361
hsa-miR-10a_000387	0,77	0,70	6,77	3,86	0,016
hsa-miR-10b#_002315	0,11	0,48	-1,62	2,62	3,328
hsa-miR-1180_002847	-8,33	3,06	-6,46	2,54	0,274
hsa-miR-1227_002769	-8,58	2,82	-6,79	2,25	0,290
hsa-miR-122_002245	4,07	0,61	4,71	0,23	0,639
hsa-miR-1244_002791	-7,73	3,70	-9,87	1,61	4,423
hsa-miR-1255B_002801	-11,34	0,13	-6,81	2,24	0,043
hsa-miR-125a-5p_002198	-2,06	0,45	-2,98	1,91	1,898
hsa-miR-125b_000449	-4,19	3,53	-5,77	3,17	2,986
hsa-miR-126#_000451	5,45	0,15	6,07	0,20	0,651
hsa-miR-1271_002779	-6,48	2,63	-9,87	1,61	10,543
hsa-miR-1274A_002883	2,14	0,56	3,72	0,66	0,334
hsa-miR-1274B_002884	8,41	0,49	8,70	0,12	0,823
hsa-miR-127_000452	1,32	0,54	-1,82	2,48	8,812
hsa-miR-128a_002216	-4,52	3,38	-2,28	2,25	0,212
hsa-miR-1290_002863	-0,93	0,77	0,18	0,91	0,462
hsa-miR-1291_002838	-8,16	3,23	-9,87	1,61	3,287
hsa-miR-1303_002792	-8,03	3,37	-9,87	1,61	3,600
hsa-miR-1305_002867	10,45	10,85	-2,92	4,87	10649,514
hsa-miR-130a_000454	3,16	0,41	3,57	0,96	0,754
hsa-miR-130b_000456	-6,71	4,50	-1,58	2,61	0,029
hsa-miR-132_000457	2,11	0,17	3,61	0,25	0,354
hsa-miR-133a_002246	2,00	0,33	-1,87	2,47	14,660
hsa-miR-133b_002247	-11,34	0,13	-6,65	3,21	0,039
hsa-miR-135a_000460	-11,34	0,13	-6,80	2,25	0,043
hsa-miR-136#_002100	-11,34	0,13	-6,47	2,53	0,034
hsa-miR-139-3p_002313	-3,53	3,86	-6,29	3,53	6,775
hsa-miR-139-5p_002289	3,43	0,12	0,10	3,44	10,074

hsa-miR-140-3p_002234	-0,37	0,75	1,30	1,11	0,314
hsa-miR-141_000463	-11,34	0,13	-5,96	3,00	0,024
hsa-miR-142-3p_000464	3,53	0,16	6,57	1,12	0,122
hsa-miR-142-5p_002248	-6,95	2,35	-2,89	1,27	0,060
hsa-miR-143_002249	-3,76	3,71	-2,21	4,38	0,342
hsa-miR-144#_002148	1,48	0,36	2,63	0,74	0,449
hsa-miR-144_002676	-7,54	3,89	3,19	1,55	0,001
hsa-miR-145#_002149	-7,96	1,84	-9,87	1,61	3,766
hsa-miR-145_002278	0,68	0,60	1,50	0,43	0,567
hsa-miR-146a_000468	7,94	0,34	8,26	0,50	0,802
hsa-miR-146b-3p_002361	-11,34	0,13	-6,81	3,08	0,043
hsa-miR-146b_001097	4,27	0,15	5,76	1,77	0,355
hsa-miR-148a_000470	1,41	0,98	3,05	0,24	0,320
hsa-miR-148b_000471	-3,61	4,00	-2,03	4,43	0,333
hsa-miR-150_000473	6,74	0,11	8,75	1,63	0,248
hsa-miR-151-5P_002642	-2,47	0,48	-1,97	1,07	0,710
hsa-miR-152_000475	0,79	0,64	-0,84	2,96	3,091
hsa-miR-155_002623	-3,61	3,84	-2,90	2,03	0,609
hsa-miR-15a#_002419	-11,34	0,13	-3,24	1,77	0,004
hsa-miR-15a_000389	0,50	0,36	2,89	0,93	0,190
hsa-miR-15b_000390	3,32	0,13	5,10	0,99	0,290
hsa-miR-16-1#_002420	-11,34	0,13	-5,94	5,51	0,024
hsa-miR-16_000391	9,08	0,47	11,13	1,21	0,241
hsa-miR-17_002308	8,40	0,19	9,35	0,89	0,518
hsa-miR-181a_000480	0,84	0,13	1,89	0,96	0,482
hsa-miR-181c_000482	-0,77	5,88	-9,87	1,61	552,246
hsa-miR-182_002334	-11,34	0,13	-8,43	1,18	0,133
hsa-miR-183#_002270	-7,29	3,92	-6,11	2,86	0,441
hsa-miR-183_002269	-8,61	2,78	-9,87	1,61	2,396
hsa-miR-184_000485	-4,12	3,75	-9,87	1,61	54,048
hsa-miR-185_002271	0,42	0,29	-1,88	2,65	4,923
hsa-miR-186_002285	4,62	0,17	5,51	1,26	0,541
hsa-miR-18a_002422	0,68	0,29	-1,36	2,70	4,102
hsa-miR-190b_002263	14,17	4,38	2,09	8,60	4322,050
hsa-miR-191_002299	7,39	0,31	8,09	0,66	0,614
hsa-miR-192_000491	1,20	0,86	4,30	0,83	0,116
hsa-miR-193a-5p_002281	1,10	0,65	1,13	0,87	0,978
hsa-miR-193b_002367	0,48	0,55	3,26	1,29	0,145
hsa-miR-194_000493	-11,34	0,13	-3,27	4,03	0,004
hsa-miR-195_000494	2,78	0,60	4,58	1,29	0,287
hsa-miR-196b_002215	-7,92	3,47	-9,87	1,61	3,866
hsa-miR-197_000497	4,76	0,14	4,13	0,35	1,543
hsa-miR-199a-3p_002304	3,57	0,30	4,52	0,20	0,519
hsa-miR-199b_000500	-8,64	2,79	-9,87	1,61	2,351
hsa-miR-19a_000395	4,54	0,41	6,50	1,19	0,258

hsa-miR-19b_000396	9,89	0,28	11,38	1,17	0,355
hsa-miR-1_002222	-1,77	1,26	-6,66	4,79	29,586
hsa-miR-200b_002251	-11,34	0,13	-5,85	3,93	0,022
hsa-miR-200c_002300	-7,69	3,70	-2,35	2,22	0,025
hsa-miR-203_000507	-11,34	0,13	-6,49	2,51	0,035
hsa-miR-204_000508	-3,94	3,64	-1,97	2,40	0,256
hsa-miR-20a_000580	8,24	0,18	9,33	1,01	0,468
hsa-miR-20b_001014	2,27	0,63	3,65	1,01	0,386
hsa-miR-210_000512	2,85	0,49	4,95	1,22	0,233
hsa-miR-214_002306	-3,57	3,84	-9,87	1,61	79,112
hsa-miR-215_000518	1,27	0,82	3,53	1,42	0,209
hsa-miR-218_000521	-8,01	3,20	-9,87	1,61	3,646
hsa-miR-21_000397	5,84	0,12	7,44	0,65	0,330
hsa-miR-22#_002301	-4,59	3,34	-2,28	2,25	0,201
hsa-miR-221_000524	6,91	0,32	6,18	0,33	1,657
hsa-miR-222_002276	5,37	0,18	6,00	0,50	0,648
hsa-miR-223#_002098	0,15	0,67	-1,98	4,38	4,365
hsa-miR-223_002295	12,97	0,12	13,59	1,00	0,652
hsa-miR-224_002099	-1,22	0,55	-5,96	3,00	26,732
hsa-miR-23a_000399	-3,64	3,81	-1,91	4,52	0,302
hsa-miR-24_000402	9,02	0,10	9,31	0,60	0,820
hsa-miR-25#_002442	-7,42	4,01	-9,87	1,61	5,481
hsa-miR-25_000403	5,01	0,16	6,52	1,06	0,352
hsa-miR-26a-1#_002443	-9,24	1,97	-7,41	1,75	0,281
hsa-miR-26a_000405	4,62	0,26	6,10	0,70	0,358
hsa-miR-26b#_002444	-7,50	2,18	-7,01	2,06	0,716
hsa-miR-26b_000407	2,84	0,35	4,80	1,12	0,257
hsa-miR-27a#_002445	-11,34	0,13	-7,30	1,83	0,061
hsa-miR-27a_000408	1,53	0,16	1,37	0,18	1,117
hsa-miR-27b_000409	7,75	2,34	6,01	2,87	3,334
hsa-miR-28-3p_002446	2,93	0,21	3,60	0,57	0,630
hsa-miR-28_000411	0,91	0,50	1,80	0,91	0,537
hsa-miR-296_000527	-0,80	1,12	-2,99	1,91	4,575
hsa-miR-29a_002112	1,56	0,26	3,75	1,00	0,219
hsa-miR-29c_000587	6,57	6,79	10,05	8,32	0,090
hsa-miR-301_000528	1,00	0,50	-5,23	3,68	75,244
hsa-miR-30a-3p_000416	-3,63	3,81	-1,48	2,77	0,225
hsa-miR-30a-5p_000417	5,29	0,28	6,53	0,64	0,422
hsa-miR-30b_000602	6,24	0,15	7,00	0,42	0,588
hsa-miR-30c_000419	6,07	0,06	6,90	0,52	0,561
hsa-miR-30d_000420	2,95	0,21	4,37	0,73	0,374
hsa-miR-30e-3p_000422	1,17	0,44	-1,62	2,58	6,923
hsa-miR-31#_002113	-11,34	0,13	-6,45	5,00	0,034
hsa-miR-31_002279	-8,11	3,28	-3,01	4,19	0,029
hsa-miR-320_002277	7,65	0,15	8,76	0,75	0,465

hsa-miR-320B_002844	-4,56	3,70	-5,95	3,01	2,612
hsa-miR-323-3p_002227	-3,93	3,63	0,62	1,65	0,043
hsa-miR-324-3p_002161	0,57	0,58	6,73	4,20	0,014
hsa-miR-324-5p_000539	-7,25	3,96	-1,97	2,41	0,026
hsa-miR-326_000542	-4,59	3,52	-6,59	2,42	4,004
hsa-miR-328_000543	4,69	0,23	4,64	0,30	1,032
hsa-miR-32_002109	-7,88	3,55	-6,32	5,13	0,339
hsa-miR-330_000544	-4,63	3,33	-3,38	1,85	0,421
hsa-miR-331_000545	2,53	0,44	3,55	0,72	0,492
hsa-miR-335_000546	2,36	0,61	-0,90	3,00	9,572
hsa-miR-339-3p_002184	0,44	0,65	-2,09	2,34	5,764
hsa-miR-339-5p_002257	-3,17	4,04	-6,04	2,93	7,317
hsa-miR-33a#_002136	-8,01	3,20	-9,87	1,61	3,644
hsa-miR-340_002258	-1,65	0,67	1,47	1,50	0,115
hsa-miR-342-3p_002260	3,28	0,21	4,74	1,27	0,363
hsa-miR-345_002186	1,29	0,49	2,44	0,55	0,449
hsa-miR-34a_000426	-11,34	0,13	-5,81	3,96	0,022
hsa-miR-361_000554	-7,45	3,76	-1,87	2,46	0,021
hsa-miR-362-3p_002117	-11,34	0,13	-6,11	2,86	0,027
hsa-miR-362_001273	-11,34	0,13	-4,78	1,31	0,011
hsa-miR-365_001020	-2,55	0,48	-1,23	0,56	0,399
hsa-miR-370_002275	-11,34	0,13	-1,80	2,51	0,001
hsa-miR-374_000563	2,83	0,45	4,59	1,18	0,296
hsa-miR-375_000564	1,54	0,53	2,36	0,99	0,565
hsa-miR-376a_000565	1,61	0,71	-0,71	3,11	4,967
hsa-miR-376c_002122	2,63	0,61	-1,10	2,84	13,219
hsa-miR-378_002243	5,18	7,19	6,53	7,79	0,393
hsa-miR-381_000571	-7,90	3,31	-9,87	1,61	3,935
hsa-miR-409-3p_002332	4,20	0,83	-0,13	3,32	20,184
hsa-miR-410_001274	1,56	0,29	-6,10	2,87	202,227
hsa-miR-411_001610	-11,34	0,13	-9,87	1,61	0,361
hsa-miR-422a_002297	-5,37	3,12	-9,87	1,61	22,761
hsa-miR-423-5p_002340	0,05	0,22	1,45	0,33	0,380
hsa-miR-425#_002302	-0,60	0,68	-2,57	2,12	3,908
hsa-miR-432_001026	-3,20	3,98	-9,87	1,61	101,864
hsa-miR-452_002329	-11,34	0,13	-6,50	2,50	0,035
hsa-miR-454_002323	1,32	0,40	3,29	1,13	0,256
hsa-miR-483-5p_002338	2,21	0,59	4,68	2,03	0,181
hsa-miR-484_001821	8,60	0,01	7,99	0,67	1,532
hsa-miR-485-3p_001277	1,07	0,61	1,64	2,07	0,676
hsa-miR-487b_001285	-4,29	3,45	-3,23	4,20	0,479
hsa-miR-494_002365	-4,00	3,60	-9,87	1,61	58,519
hsa-miR-501-3p_002435	-11,34	0,13	-6,17	2,81	0,028
hsa-miR-502-3p_002083	-8,25	2,97	-9,87	1,61	3,093
hsa-miR-505#_002087	-5,35	3,24	-6,75	2,28	2,648

hsa-miR-505_002089	-7,74	3,65	-6,35	2,64	0,381
hsa-miR-512-3p_001823	-7,56	3,83	-9,87	1,61	4,969
hsa-miR-517a_002402	-11,34	0,13	-6,96	2,95	0,048
hsa-miR-532-3p_002355	0,47	0,21	-3,26	1,93	13,255
hsa-miR-532_001518	2,07	0,90	4,76	0,97	0,154
hsa-miR-539_001286	-7,82	3,39	-6,02	2,94	0,287
hsa-miR-542-5p_002240	-7,99	3,44	-9,87	1,61	3,703
hsa-miR-543_002376	-7,16	4,05	-7,03	2,05	0,918
hsa-miR-574-3p_002349	4,03	0,28	4,08	0,15	0,969
hsa-miR-576-3p_002351	-8,09	3,31	-6,56	2,46	0,347
hsa-miR-579_002398	-7,98	3,23	-9,87	1,61	3,711
hsa-miR-590-3P_002677	-5,97	2,65	-1,87	0,74	0,058
hsa-miR-590-5p_001984	1,88	0,46	3,37	1,94	0,354
hsa-miR-598_001988	-4,77	3,42	-2,33	2,26	0,184
hsa-miR-605_001568	-5,98	2,82	-9,87	1,61	14,889
hsa-miR-624_001557	-11,34	0,13	-6,23	2,75	0,029
hsa-miR-625#_002432	2,49	0,44	1,51	1,32	1,966
hsa-miR-625_002431	-5,18	3,04	-6,86	2,20	3,199
hsa-miR-627_001560	2,21	10,35	-9,87	1,61	4338,057
hsa-miR-628-3p_002434	-2,05	0,40	3,79	11,45	0,017
hsa-miR-628-5p_002433	2,54	9,55	-9,87	1,61	5460,561
hsa-miR-629_001562	-7,83	3,60	-6,40	5,05	0,371
hsa-miR-629_002436	-8,21	3,18	-9,87	1,61	3,161
hsa-miR-636_002088	8,67	3,95	10,88	5,74	0,217
hsa-miR-642_001592	-8,07	3,32	-6,71	3,16	0,388
hsa-miR-645_001597	-4,80	3,20	-9,87	1,61	33,610
hsa-miR-652_002352	0,93	0,13	2,95	1,34	0,248
hsa-miR-655_001612	-4,88	3,40	-7,63	1,59	6,750
hsa-miR-656_001510	-11,34	0,13	-6,30	5,15	0,030
hsa-miR-660_001515	2,38	0,73	5,26	0,81	0,136
hsa-miR-661_001606	-2,34	4,63	-1,30	4,78	0,486
hsa-miR-671-3p_002322	-0,93	0,39	-6,53	2,48	48,666
hsa-miR-708_002341	-7,69	3,53	-9,87	1,61	4,560
hsa-miR-744_002324	-0,31	0,48	-5,70	3,24	42,015
hsa-miR-766_001986	3,02	0,13	-0,42	3,17	10,839
hsa-miR-769-5p_001998	-0,97	0,38	1,53	1,15	0,177
hsa-miR-885-5p_002296	3,82	0,69	0,29	3,53	11,531
hsa-miR-886-3p_002194	-7,63	3,77	-9,87	1,61	4,753
hsa-miR-888_002212	14,56	0,47	16,00	1,27	0,368
hsa-miR-9#_002231	-11,34	0,13	-2,70	4,23	0,003
hsa-miR-92a_000431	8,20	0,35	9,12	0,81	0,528
hsa-miR-93#_002139	-4,61	3,30	1,05	0,86	0,020
hsa-miR-942_002187	-7,21	4,22	-1,70	2,59	0,022
hsa-miR-943_002188	-7,47	3,74	-9,87	1,61	5,282
hsa-miR-95_000433	-11,34	0,13	-2,89	2,06	0,003

hsa-miR-99a_000435	-7,87	3,56	-6,00	3,79	0,274
hsa-miR-99b#_002196	-7,57	3,64	-2,92	3,98	0,040
hsa-miR-99b_000436	0,93	0,16	-0,40	5,19	2,503
mmu-miR-134_001186	-0,16	1,08	-3,44	1,68	9,728
mmu-miR-140_001187	2,73	0,21	4,08	1,22	0,393
mmu-miR-374-5p_001319	2,56	0,22	3,11	0,76	0,685
mmu-miR-451_001141	7,05	0,50	9,87	1,49	0,142
mmu-miR-491_001630	-3,89	3,65	1,71	1,38	0,021
mmu-miR-495_001663	1,69	0,89	-1,84	2,58	11,550
mmu-miR-93_001090	4,99	0,38	6,13	1,02	0,455
rno-miR-29c#_001818	-11,34	0,13	-7,66	1,56	0,078
rno-miR-7#_001338	2,86	0,20	3,38	1,31	0,695
U6 rRNA_001973	3,37	0,58	5,32	2,11	0,259

Circulating miRNA were detected by Open Array plate in plasma of CAD patients (n=3) and control subjects (n=3). Data are shown as relative miRNA expression levels and normalized using the global mean normalization method and expressed as -△Ct. The expression fold change are expressed as 2^(△△Ct) in which values represent the fold change miRNA expression of CAD patients relative to miRNA expression of control subjects.
